# Dementia care in public health in Brazil and the world: A systematic review

**DOI:** 10.1590/S1980-57642014DN81000007

**Published:** 2014

**Authors:** Bianca Bolzan Cieto, Gabriela Gallego Valera, Glaucia Bueno Soares, Roberta Hehl de Sylos Cintra, Francisco Assis Carvalho Vale

**Affiliations:** 1 Nurse. Master's degree in Nursing by the Graduate Program in Nursing at the Federal University of São Carlos - UFSCar. São Carlos, São Paulo, Brazil.; 2 Nurse. Master's degree in Nursing by the Graduate Program in Nursing at the UFSCar. São Carlos, São Paulo, Brazil.; 3 Nurse. Master's degree in Nursing by the Graduate Program in Nursing at the UFSCar. São Carlos, São Paulo, Brazil.; 4 Lawyer, Biomedical. Master's degree in Ecology and Natural Resources Graduate Program at the UFSCar. São Carlos, São Paulo, Brazil.; 5 Neurologist. Adjunct Professor of the Department of Medicine of the UFSCar. São Carlos, São Paulo, Brazil.

**Keywords:** dementia, public health, health policy

## Abstract

**Objective:**

This study aimed to identify in the recent scientific literature, information on health care provided to people with dementia, dementia costs and its resource implications for public health.

**Methods:**

This was a systematic review of the literature in which the articles were consulted from the databases PubMed/MEDLINE, LILACS and SciELO. The review sample consisted of 45 articles.

**Results:**

Examination of the studies identified the current scenario of dementia in relation to public health and public policy in Brazil and the world. The analyzed studies revealed key information on aspects of dementia in the world. There was consensus on the high prevalence of the syndrome and on the significant cost of health care and public policy for assisting the elderly with dementia.

**Conclusion:**

The importance of planning and implementing new public policies was recognized, since these are essential for the organization and management of health services and directly influence the country's ability to provide health care for people with dementia.

## Abstract (pt)

**Objetivo:**

Este estudo objetivou identificar na literatura científica recente, informações sobre a atenção à saúde proporcionada à pessoa com demência, os custos com demência e sua implicação nos recursos destinados a saúde pública.

**Métodos:**

Trata-se de uma revisão sistemática da literatura em que foram consultados artigos das bases de dados PubMed/MEDLINE, LILACS e SciELO. A amostra da revisão constituiu-se de 45 artigos.

**Resultados:**

A análise dos estudos possibilitou identificar o contexto atual da demência em relação a saúde pública e políticas públicas, no Brasil e no mundo. Observou-se que os estudos analisados trouxe aspectos importantes informativos sobre a demência no mundo. Houve um consenso de que a prevalência desta síndrome é elevado, assim como o custo dos cuidados de saúde e da política pública é relevante para a saúde do idoso com demência.

**Conclusão:**

Concluiu-se que há pertinência e necessidade de planejar e implementar novas políticas públicas, uma vez que são fundamentais na estruturação e gerenciamento de serviços de saúde e influenciam diretamente na capacidade do país prover atenção à saúde as pessoas com demência.

## INTRODUCTION

Population aging is occurring worldwide and has promoted a growing increase in the occurrence of health problems such as dementia. The rise in the number of people with dementia also results in a growing increase of healthcare costs.^1,2^

The dementias, predominantly resulting from chronic and progressive neurodegenerative diseases, are associated with serious mental and physical disorders that interfere with a person's life, as well as that of their families. These conditions are debilitating and often affect the lives of family members, who have to dedicate time to caring for the patient, and often cause anxiety and depression in caregivers.^2^ According to the World Health Organization (WHO), there are currently 35.6 million people living with dementia worldwide, a number set to double by 2030 and more than triple by 2050.^3^

The high prevalence, social exclusion and the economic impact of dementia in families, caregivers and communities affect both developed and developing countries and constitute a major public health problem that requires consistent governmental actions, as declared by the WHO and the Alzheimer Disease International.^3,4^

Dementia care costs are increasing rapidly, especially in developed countries, but also in developing nations, and the needs of elders with dementia is expected to progressively impact health and social services budgets.^4^

One in every three seniors may have dementia, and their caregivers, mostly relatives, suffer a severe psychological and physical overload due to the role they play. In most countries, the available services for catering to needs of this group are insufficient, especially in developing countries.^5^

Therefore, reorientation and changes in public policy for appropriate assistance is necessary, investing primarily in basic care with new approaches for preventive strategies and health promotion. Health professionals, especially those working in primary care settings should be the target of ongoing training to cater for the needs of elderly with dementia.^6^

This systematic review aimed to identify, in recent scientific literature, information on health care provided to people with dementia in developed and developing countries, the impact of the increasing costs of dementia on public health resources, and determine healthcare requirements.

## METHODS

A bibliographic review was carried out in order to identify topics related to the attention given to dementia in public health of developed and developing countries, spending on dementia and its impact on financial resources, and the requirements for provision of adequate healthcare to individuals with dementia.

The active search of information was performed using the databases LILACS, SciELO and PubMed/MEDLINE. The descriptors were taken from MeSH and DeCS in English and used, along with their equivalents in Portuguese and Spanish, in the following combinations: "*public health* AND *dementia* AND *aged* AND *developed countries* OR *developing countries"; " public health* AND *dementia* AND *developed countries* OR *developing countries"; " public health* AND *dementia* AND *aged, 80 and over* AND *developed countries* OR *developing countries"; " public health* AND *dementia"; " public health* AND *dementia* AND *aged"; " health policy* AND *dementia* AND *developed countries* OR *developing countries"; " health policy* AND *dementia*".

Articles indexed in these databases from January/2007 to July/2012 and written in Portuguese, English or Spanish were considered inclusion criteria. Exclusion criteria were not used for the initial search. Submission of the study to the Committee of Research Ethics Committee was not necessary because this constituted a review of the literature.

In the first step, a single search was made of the databases and articles were selected by title and summary apparently related to the theme. As a second step, the articles were examined by the author, and any duplicates were deleted. Subsequently, the studies were divided among the authors to be fully read. As a third step, the authors conducted a review of these articles and those relevant to the subject of this work were retained.

## RESULTS

Through the intersection of descriptors in the databases, 654 articles were identified in PubMed/MEDLINE, 67 in the LILACS and 25 in SciELO databases. After reading all the titles and abstracts, 646 articles, were excluded due to the absence of relationship with the subject while the remaining 100 were taken in full. After reading these articles, 45 were selected as relevant to the topic and studied for this review (Figure).

**Figure:**
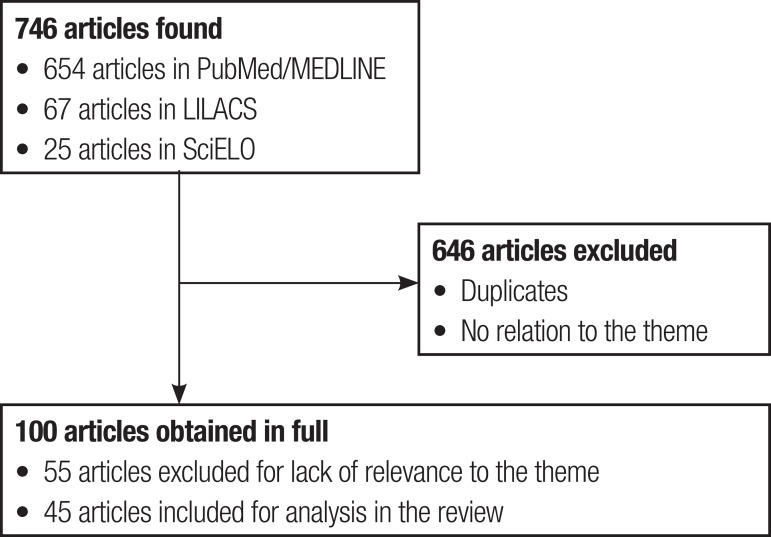
Flowchart with sample selection steps forming this systematic review.

In reference to type of study, 14 were non-systematic reviews with expert opinion, 13 were cross-sectional, population-based, 7 were case-by-case studies, 4 compiled data analyses, 3 systematic reviews, 2 population-based cohorts, and 2 randomized controlled clinical trials.

The study covered 197 countries distributed over five continents. of which 22 studies involved countries in the Americas, 15 countries in Europe, 9 in Asia, 6 in Africa and four studies in Oceania.

Twenty-seven studies indicated needs for healthcare assistance in people with dementia, 20 discussed ways in which developing and/or developed countries promote this care and 5 reported data concerning costs and impacts on financial resources related to dementia.

Regarding the journals in which the studies were published, 30 were published in interdisciplinary journals in the area of health, 13 in medical journals and two in public health journals.

A summary of the data extracted from the articles is presented in descriptive form in the Table.

**Table:** Articles included in the review by author, year, type, continent/country and approach.

Author/Year	Type	Continent/Country*	Approach
Chaufan C, et al.^7^2012	Review, expert opinion	United States	Evolution of the concept of AD**, the emergence of social movements and offering of alternatives
Faure-Delage A, et al.^8^2012	Population-based, cross-sectional	Congo	Cultural representations of dementia and their knowledge in Brazzaville
Mitchell SL, et al.^9^2012	Review, expert opinion	United States	Points out the difficulties faced in the management of dementia and indicates solutions and possible alternatives
Toure K, et al.^10^2012	Population-based, cross-sectional	Senegal	Alert the community about the prevention of dementia
Sosa AL, et al.^11^2012	Population-based, cross-sectional	Cuba, Dominican Republic, Peru, Mexico, Venezuela, Puerto Rico, China, India	Health cost implications caused by the increased incidence of dementia
Maestre GE^12^2012	Non-systematic review	Venezuela	Professional training, public education, financial, ethical and infrastructure resolutions
McParland P, et al.^13^2012	Population-based, cross-sectional	Northern Ireland	Evaluates the knowledge about dementia to improve the educational public health policies
Llibre JJ, et al.^14^2011	Population-based cross-sectional	Cuba	The absence of health policy in communicable diseases and dementia
Abbott A^15^2011	Expert opinion	Germany	Economic costs of dementia and care programs based on data from Alzheimer's Disease International (ADI)
Carias CM, et al.^16^2011	Analysis of compiled data	Brazil	Program costs of dispensing medicines in exceptional situations
Rosow K, et al.^17^2011	Systematic review	European Union (27 countries), Canada, Australia, China, United States, India, South Korea	National plans for programs in prevention, research and treatment of dementia diseases
Gustavsson A, et al.^18^2011	Systematic review	Europe	Recommends an action policy for the high cost of brain disorders
Wittchen HU, et al.^19^2011	Systematic review	European Union (27 countries), Switzerland, Iceland, Norway	Increased funding in order to identify best strategies for prevention and treatment
Luengo-Fernandez R, et al.^20^2011	Analysis of compiled data	European Union (15 countries)	Demonstrates that dementia has a significant cost for health and society in general
Guerra M, et al.^21^2011	Randomized clinical trial	Peru	Intervention with caregivers of people with dementia reduces the tension of the their role
Jacinto AF, et al.^22^2011	Cross-sectional, case-by-case study	Brazil	Points out that resident physicians rarely identify elderly with cognitive decline
Rapp T, et al.^23^2011	Population-based, cross-sectional	France	Public financial support to people with dementia as a stimulus for informal networks of care
Miranda LFJR, et al.^24^ 2011	Cross-sectional, case-by-case study	Brazil	Identification of the most probable causes of late diagnosis
Wimo A, et al.^25^2010	Analysis of compiled data	Latin America, Africa, Europe, Oceania, Asia, North America	Cost of dementia based on prevalence, ADL**, GDP** and cost per person
Brosselin P, et al.^26^2010	Analysis of compiled data	France	Underreporting of deaths from Alzheimer's or dementia on death certificates
Awata S^27^2010	Review, expert opinion	Japan	History of health and incidence of dementia. Foundation of the Dementia Center for Senior Citizens
Todd S, et al.^28^2010	Expert opinion	Northern Ireland	Developing health services and strategies to support people with dementia in Ireland
Cahill S^29^2010	Expert opinion	Ireland	Identified priorities by the main social action plan on dementia and their weaknesses
Castro DM, et al.^30^2010	Review, expert opinion	South America	Evaluation of costs and notes on possible solutions for optimization
Larson EB^31^2010	Non-systematic review	United States	Dementia at the end of life is increasingly common and poses a challenge to people, caregivers and public health
Wang Y, et al.^32^2010	Population-based cohort,	China	Survival time of patients with dementia and the construction of a tool to classify type of dementia
Banerjee S^33^2010	Expert opinion	England	Methods to solve the lack of assistance and support to the family and the person with dementia
Prince MJ^34^2009	Population-based, cross-sectional	Argentina, Venezuela, Peru, Brazil, Mexico, Cuba, China, India, Nigeria, South Africa	Need for knowledge about dementia and health policies and specialized services
Sowmini CV, Vries R.^35^2009	Non-systematic review	Netherlands, India	Parallel study on the differences in approaches of people with dementia in the Netherlands and India
Travers C, et al.^36^2009	Review, expert opinion	Australia	Barriers and facilitators to health promotion, prevention and early intervention in dementia
Llibre JJ, et al.^37^2009	Population-based, cross-sectional	Cuba	Dementia and Alzheimer's disease is a major and growing health problem in Cuba
Guerchet M, et al.^38^2009	Population-based cross-sectional	Benin	Prevalence of dementia in elderly living in rural areas of Benin and the need for further studies in Africa
Justiss MD, et al.^39^2009	Cross-sectional, case-by-case study	United States, United Kingdom	Screening for dementia as an important part of the process of early identification
Nitrini R, et al.^40^2009	Population-based cohort	Latin America	The lack of access to basic care and the different diagnostic methods making prevalence vary
Uwakwe R, et al.^41^2009	Population-based, cross-sectional	Nigeria	The types of health care and contributions made by the Government and by patients
Prince MJ, et al.^42^2008	Non-systematic review	India, Brazil, United Kingdom, China, Russia	The management of dementia in peripheral countries and the effectiveness of interventions.
Dias A, et al.^43^2008	Randomized controlled trial	India	Intervention improves the quality of caregivers' lives and reduces mortality in people with dementia
Magalhães MOC, et al.^44^2008	Population-based, cross-sectional	Brazil	Methods of prevention and control of risk factors, which can reduce the prevalence of dementia
Pavarini SCI, et al.^45^2008	Cross-sectional, case-by-case study	Brazil	Use of an information system, integrating geography and health care in planning and management of public health programs
Fuh JL, Wang SJ.^46^2008	Review, expert opinion	Taiwan	The necessity of a local agreement for the diagnosis and treatment of dementia in Taiwan
Scazufca M, et al.^47^2008	Population-based, cross-sectional	Brazil	Prevalence of dementia is high among the elderly with low socioeconomic level
Brunton M, et al.^48^2008	Cross-sectional, case-by-case study	New Zealand	Subjects considered within health policies in order to improve caregivers' support
Lopes MA, et al.^49^2007	Population-based, cross-sectional	Brazil	Elderly people with dementia should receive more attention in public health policies
Purandare N, et al.^50^2007	Cross-sectional, case-by-case study	United Kingdom	Socio-cultural barriers and lack of knowledge about dementia affects the search for care
Allegri RF, et al.^51^2007	Cross-sectional, case-by-case study	Argentina	Direct and indirect economic costs with Alzheimer's in Argentina, compared to other studies

* In review and/or expert opinion studies, the country of origin was considered the country of the author(s);

** AD Alzheimer's disease; ADL Activities of Daily Living; GDP Gross Domestic Product.

## DISCUSSION

The analyzed studies revealed key information on various aspects of dementia in the world. There was a consensus on the high prevalence of this syndrome as well as on the substantial cost of health care and public policies for assisting the elderly with dementia.

In some countries, actions involving health care for the elderly with dementia are carried out through programs or national plans, which aim to reduce risk factors, raise awareness, assist with caregivers, promote health education activities, provide early diagnosis, and condition-assessment tools and technologies that support the management of public health problems. All of these initiatives seek to improve the identification of health needs, provide for appropriate and effective care planning, social support and family awareness of the population in general.

It is understood that recognizing the need to improve and develop instruments, technologies and public policies for healthcare of elderly people with dementia, fosters reflective-critical analyses among policy makers and health managers on the weaknesses of the country. This process legitimizes the context of daily practices and proposes improvements to the country.

Some national plans for the prevention and treatment of dementia are presented by developed and developing countries,^13,17,23,29,37,48^ which focus on ensuring rights and enhancing the quality of life of elderly people with dementia and their close ones.^33^

The presumption that only developed countries promote health care plans has not been confirmed in this review, since some developing countries also work toward this goal. Nevertheless, it should be noted that developed countries tend to be more advanced in health care of dementia, since other countries suffer from poor allocation of formal resources, unequally distributed around the world.

It is estimated that the total world cost of dementia was $422 billion in 2009,^25^ which represents a significant burden for health, social services and society in general.

As a representation of the financial burden generated by dementia, the total cost of dementia in Europe has been estimated at €189 billion a year, of which €49 billion is spent on social assistance services and €10 billion covers health costs. This represents an annual cost per elderly with dementia of approximately € 10,000 to social and health services, while the unpaid care for family members (informal care) is € 120 billion of total costs. As dementia occurs predominantly in the elderly, the loss of productivity, morbidity and mortality are not considered significant, however, if morbidity cost were estimated as working days lost due to the impact of the syndrome, productivity loss would exceed €7.3 billion.^20^

There is no assessment of the cost of dementia in Brazil, but a study showing the costs of the free dispensing of medicines for a variety of dementia syndromes, estimates spending of approximately R$307 million in 2007.17 However, as in other countries, the limited role of the Brazilian government in social and financial support, places the onus on the family as the main carer and supporter of the elderly.^41^

Brazil has government healthcare programs and policies focusing on the elderly population in order to promote physical, mental and social wellbeing. Nevertheless, there are many problems in the implementation of these policies and programs, financial fundraising and in providing adequate training of human resources. This shows a weak sociopolitical scenario which lacks effective cooperation among the three levels of SUS management (Federal, State and Municipal).^52^

It is important to mention that despite this difficulty, the proposals contained in public policies and health programs redirect attention to the health of the elderly with dementia and their families, in that they formalize the health needs of this specific population and contribute to the development of new models that reduce the social and economic cost of dementia.

The economic and political organization of developed countries enables them to plan and provide the population with appropriate health services which are constantly improved, as well as foster the social support networks consolidated by them.^27-28,48^ In addition, financial aid for elderly people with dementia helps reduce the burden placed on the informal caregiver, increasing the quality of life of both parties and delaying institutionalization.^23^

It is understood that another way to assure the elderly quality of life is through research funding^18-19^ because such research presents the reality experienced, investigates causes of dementia syndromes and ways to develop medicines, prevention and treatments.

In developing countries, the political fabric tends to be inadequately structured, the financial resources for the needs of the country insufficient, and the management of public policies ineffective.

Public policies are recognized as essential for assessment of the impact of dementia on resources and health spending, and their strengthening guarantees the identification of prevalence, risk factors, diagnosis and treatment, social support and quality of life for the elderly and their caregivers.^29,30,33,38,43,46^ All of these are relevant points to reduce the financial impacts^16^

Due to the lack of public policy in many countries, low-cost strategies and positive health outcomes have been developed with the purpose of compensating for the absence of formal services and support networks. Interventions with the caregiver, guidance, dissemination and socialization of knowledge about dementia, all improve the quality of life of the people involved in this context.^21,43^

Accordingly, Non-Governmental Organizations (NGOs) and research groups from academic institutions are a strategy commonly used by low and middle-income countries. In Brazil, the Brazilian Association of Alzheimer (ABRAz) is a nationwide NGO known in welcoming and supporting families affected by dementia.

The universities are considered equipped to meet the needs of the elderly population. These institutions can promote cognitive and social activities for the elderly, dissemination of information to society about dementia and deliver health education to caregivers. Also, they contribute to the production of research knowledge and in the training of more qualified professionals.^53^ It is noteworthy that essential health professionals are properly trained on dementia issues.^18,19,22,29^

Thus, these actions are another component of an integrated network, which together govern different areas to promote targeting of new models of healthcare actions and strengthen comprehensive care to the elderly with dementia and their families.

In conclusion, the use of a systematic review method to seek information available in the literature concerning dementia issues in public health of developed and developing countries has enabled us to identify the latest knowledge on the subject.

A global scientific output has been identified, which contextualizes the issues on dementia in order to foster reflection and discussion to raise awareness among political leaders, health professionals and society at large on the high costs and impact on public health resources. The review highlights the considerable extent to which health, social and government needs have not yet been met, in addition to the importance of funding and promoting studies to improve prevention and treatment strategies.

Moreover, it was confirmed that the presence of public policies positively influences the ability of the country to provide care for its elderly population with dementia, as they ensure quality assistance and support with the management of public resources. It is also evident that the activities carried out by NGOs and universities complement the healthcare actions in dementia and together with public policies can reduce cost to the State.

Thus, the results of this review reaffirm the commitment that policy makers and public policy managers have in decision-making regarding dementia, given the major social, financial and health impacts on the elderly, their families and society as a whole.
